# Autologous bone marrow-derived cell transplantation in decompensated alcoholic liver disease: what is the impact on liver histology and gene expression patterns?

**DOI:** 10.1186/s13287-017-0541-2

**Published:** 2017-04-18

**Authors:** Nicolas Lanthier, Nathalie Lin-Marq, Laura Rubbia-Brandt, Sophie Clément, Nicolas Goossens, Laurent Spahr

**Affiliations:** 1 Gastroenterology and Hepatology, University Hospitals and Faculty of Medicine, 4, Rue Gabrielle Perret-Gentil CH-1211, Geneva 14, Switzerland; 2 Laboratory of Gastroenterology and Hepatology, Institut de Recherche Expérimentale et Clinique, Université catholique de Louvain, Brussels, Belgium; 3 Clinical Pathology, University Hospitals and Faculty of Medicine, Geneva, Switzerland

**Keywords:** Stem cell transplantation, Macrophage, Kupffer cell, SPINK1, CD68, HGF, Alcoholic hepatitis, Cirrhosis

## Abstract

**Background:**

Liver stem cell therapy (SCT) has been suggested as a promising means to improve liver regeneration in advanced liver disease. However, data from trials are heterogeneous, with no systematic histological evaluation. The aim of this study is to specifically analyze the effect of autologous SCT on liver regeneration and on gene expression changes.

**Methods:**

Individuals in the randomized controlled trial of SCT in alcoholic hepatitis with paired liver biopsies were included (*n* = 58). Immunohistochemistry (Ki67, K7, and CD68), in situ hybridization (SPINK1), and global gene expression analysis were performed on liver biopsies (30 control patients and 28 patients with transarterial administration of bone marrow-derived stem cells) both at baseline and after 4 weeks of follow-up.

**Results:**

No difference between the two groups could be observed regarding the proliferative hepatocyte number, proliferative K7-positive cells, or total K7-positive cells at the 4-week follow-up liver biopsy. However, patients who received SCT showed a more important liver macrophagic expansion as compared to standard treatment. Transcriptome data revealed changes in genes linked with inflammation (CD68 and SAA), regeneration (SPINK1 and HGF), fibrosis (COL1A1), and stem cells (CD45). No changes in gene pathways involved in liver growth and cell cycle proteins were evident. SPINK1 mRNA was present by in situ hybridization at week 4 in SCT patients in the liver parenchyma areas adjacent to macrophage recruitment and liver cell proliferation.

**Conclusions:**

The analysis of liver tissue after SCT demonstrated an expansion of macrophages concurrent with an upregulated expression of genes involved in inflammatory and regenerative pathways. With the negative results from the clinical trial, the impact of the SCT has to be interpreted as weak, and it is not able to modify the clinical course of this severe liver disease.

**Electronic supplementary material:**

The online version of this article (doi:10.1186/s13287-017-0541-2) contains supplementary material, which is available to authorized users.

## Background

There is a great interest in stem cell therapy (SCT) in the setting of an increased burden of chronic decompensated liver diseases and an organ shortage for liver transplantation. Accordingly, decompensated alcoholic liver disease (ALD) with alcoholic hepatitis (AH) carries a poor prognosis with elevated short-term mortality even in steroid-treated patients. Early liver transplantation can be an option in very select patients, but this treatment strategy is unavailable to the majority of patients [1].

Stem cell-based strategies have been tested as an alternative to organ transplantation. However, limitations of clinical trials that aimed to explore the effect of granulocyte-colony stimulating factors (GCSF) [2, 3] and/or stem cell transplantation [4, 5] include lack of power due to the small sample size and major heterogeneity in the protocol design (including absence of systematic liver biopsy), disease etiologies, endpoints, methods of stem cell collection (from the bone marrow or from the blood, with or without cell mobilization by GCSF), cell infusion number, and route of administration. The results of those investigations are summarized in two recent reviews [6, 7]. In particular, systematic investigations of SCT in AH (controlled and histologically based) are of great importance with regards to the heterogeneity of the disease, the role of alcohol abstinence, and the possible impact on specific pathways.

In a recent randomized controlled trial [8], identified as a high-quality study [6], we investigated the effect of autologous bone marrow stem cell transplantation (SCT) in AH and explored the evolution of histological alterations during follow-up using a repeat liver biopsy at 4 weeks. Although our trial did not demonstrate any clinical benefit in terms of the model for end-stage liver disease (MELD) score changes over a 3-month period, nor any differences in progenitor cell proliferation by immunohistochemistry on repeat liver biopsy [8], we felt that further investigations were needed on liver histology and on hepatic gene expression. In particular, the impact of SCT on proliferating hepatocytes, total K7 progenitor cells, and also hepatic macrophages remained to be analyzed. Indeed, macrophages constitute a heterogeneous population within the liver, both promoting detrimental inflammation and also being involved in regenerative response after injury, and have been identified as good prognosis markers [9–11]. Actually, previous results highlighted that improvers (patients with a significant improvement of liver function defined by a decrease in MELD score of 3 points or more at 3 months) were characterized at baseline by a significantly higher number of proliferating hepatocytes, proliferative K7 progenitor cells, and higher macrophage infiltration compared to nonimprovers (defined as patients with MELD deterioration or MELD improvement lower than 3 points at 3 months or patients who died during this 3-month follow-up).

Thus, the aim of this study was to perform an in-depth histological and transcriptome analysis both at baseline and at repeat liver biopsy performed after 4 weeks in order to detect whether SCT was associated with specific changes in macrophages and regenerative pathways.

## Methods

### Patients and tissue collection

Data for this study were obtained from participants in the autologous SCT in decompensated alcoholic liver disease trial [8]. Written informed consent was obtained in all cases and the study was accepted by our institution’s ethics committee. According to recent guidelines [12], the syndrome of AH was defined by a recent clinical decompensation (ascites and/or jaundice) in active drinkers (≥80 g/day until admission) and exclusion of other causes of decompensation such as ongoing infection, hepatocellular carcinoma, biliary tract obstruction, gastrointestinal bleeding, and portal vein thrombosis. Liver biopsy was performed by the transjugular route in all patients early after hospital admission and prior to specific therapeutic interventions. In all patients, liver histology showed established cirrhosis with superimposed acute lesions including steatosis. Histological lesions were evaluated by a blinded pathologist on hematoxylin and eosin stained histological slides using a semiquantitative scoring of steatosis, portal and lobular inflammation, and hepatocyte ballooning. After screening for those clinical and histological criteria, 58 patients with AH were included and divided into two groups: one (control) group received only standard of care management and another group received a single session of autologous SCT (CD34^+^ stem cells and mesenchymal stem cells collected the same day from the bone marrow) into the hepatic artery following mobilization using a 5-day course of subcutaneous GCSF in addition to standard of care therapy (Table 1). Cell isolation and characterization procedures are described in our first report [8]. The mean number of bone marrow-derived cells infused into the hepatic artery was 0.47 ± 0.15 × 10^8^/kg, within the range seen in other studies [13]. Standard of care management included vitamin B supplementation, renutrition, alcohol withdrawal, and treatment with prednisone 40 mg/day for 4 weeks when indicated (Maddrey discriminant function ≥32 and histologically proven alcoholic steatohepatitis). A repeat liver biopsy was performed at 4 weeks, and follow-up information was carefully collected over 3 months. As for the first biopsy, the liver tissue was separated into two pieces, one for histological analysis and the other snap-frozen in liquid nitrogen and stored at –80 °C for further gene expression analysis. Six patients died during the study (four in the control group and two in the SCT group). Improvement of liver function during follow-up as assessed by the MELD score was similar in the two groups and no differences could be observed in histological lesions between the two groups using hematoxylin and eosin staining.

**Table 1:** Patient baseline inclusion characteristics according to treatment allocation

Variable	Control patients (*n* = 30)	SCT patients (*n* = 28)	*p* value
Age (years)	57 (37–69)	54 (35–67)	0.15
Gender (M/F)	19/11	14/14	0.31
HVPG (mmHg)	19.9 ± 2.4	19.1 ± 2.8	0.28
MELD score	19.1 ± 4.0	19.0 ± 3.8	0.86
Cirrhosis (%)	100	100	0.99
ASH (n, %)	25 (83.3%)	22 (78.6%)	0.64
Corticosteroid treatment (n, %)	22 (73.3%)	16 (57.1%)	0.59

*ASH* alcoholic steatohepatitis (full histological definition), *F* female, *HVPG* hepatic venous pressure gradient, *M* male, *MELD* model for end stage liver disease, *SCT* stem cell therapy

### Immunohistochemistry

All liver biopsy specimens (*n* = 58 at baseline, *n* = 52 at 4 weeks) were processed as previously described [10]. Briefly, after fixation into formalin 10% and paraffin embedding, immunostainings were performed using the automated Ventana Discovery XT system (Ventana Medical Systems, Tucson, AZ, USA) and Ventana reagents. The mouse monoclonal human antibody MIB1 against the proliferation marker Ki67 (Dako, M7240, 1:100 dilution), anti-Keratin7 (K7; Dako, M7018, 1:100 dilution), and anti-CD68 (Dako, M0876, 1:100 dilution) were used as primary antibodies and detected.

Proliferation was assessed on the whole biopsy specimen by manual counting under very high magnification (×400). Results are given as the mean number of Ki67-positive cells per high-power field. Ki67-positive cells were classified either as K7-negative or -positive to assess either hepatocyte (Hep) or liver progenitor cell (LPC) proliferation. K7-positive cells were further classified by their histological appearance, localization in the liver lobule, and staining intensity of K7. Cells from the ductular reaction (DR) are cuboid adjacent K7 intense positive cells forming ductules. Intermediate progenitor cells (iPC) are K7-positive cells (often isolated) located in the liver lobule and are smaller than hepatocytes. Intermediate hepatocytes (IH) are large, cuboid hepatocyte-like cells with a less intense K7 staining. Total K7 and CD68 (a marker for liver macrophages) positive cell areas were quantified using tissue section photographs at high magnification (×100) of the entire liver biopsy and using the Leica QWin computer assisted program [14]. Results are given as the percentage of total positive cell area (K7 or CD68) on the total biopsy surface at × 100 magnification. As for hematoxylin and eosin staining evaluation, immunohistochemistry was analyzed in a blinded manner.

### In situ hybridization

The PCR template coding partially for human serine peptidase inhibitor Kazal type 1 (SPINK1) was generated from human pancreas RNA (AM7954, AMBION) using the reverse transcription kit from Takara (RR037A), and SPINK1 in situ hybridization with sense or antisense probes specific for human SPINK1 was performed on serial sections from paraffin-embedded human liver, as previously described [10], with human pancreas tissue sections serving as positive controls.

### Transcription analysis

Transcriptome profiling was performed with Affymetrix arrays containing 49,395 transcripts. Total RNA was extracted from snap-frozen liver biopsies obtained at baseline (*n* = 30) and after 4 weeks (*n* = 24) using the TissueLyser machine (Qiagen) and the AllPrepDNA/RNA Mini Kit (Qiagen, Hombrechtikon, Switzerland). RNA quality was assessed by capillary electrophoresis on the Agilent 2100 Bioanalyzer (Agilent Technologies, Basel, Switzerland); 100 ng was amplified and labeled using the 3’ IVT Express kit (Affymetrix). Hybridization on GeneChip PrimeView Human Gene Expression arrays (Affymetrix) was carried out according to the manufacturer’s instructions.

The data were robust multi-array normalized [15]. Pathway analysis of the genes, which were identified as differentially expressed by the microarray experiment, was undertaken using the MetaCore software (http://www.genego.com). The gene expression data can be found in ArrayExpress (https://www.ebi.ac.uk/arrayexpress/) accession no. E-MTAB-2664.

### Nanostring

For the confirmation of the Affymetrix transcriptional analysis, 27 samples were assessed using the nCounter system (NanoString). NanoString synthesized the codeset for the analysis of 115 genes of interest and 7 normalization genes. RNA hybridization, sample processing, and calculation methods were performed as previously described [10].

### Statistical analysis

Continuous variables are presented as means ± SD and were compared using Student’s *t* test (two-tails) or paired if appropriate. Categorical variables were compared using the chi square test. To assess the differences in gene expression values (Affymetrix and Nanostring) between the different groups (controls versus SCT at 4 weeks of follow-up, follow-up versus baseline in each group), we performed a 5-way analysis of variance (ANOVA) with contrast in Partek Genomics Suite (http://www.partek.com). We applied a significance threshold *p* value of 0.05.

## Results

### Effect of SCT on liver histology

**Fig. 1:**
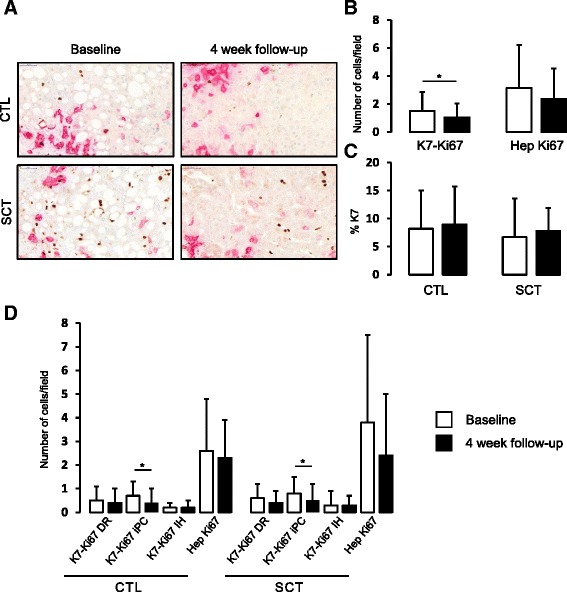
Liver progenitor cell compartment analysis at baseline and 4 weeks in controls (*CTL*) and stem cell treated (*SCT*) patients. Total liver progenitor cells were identified by keratin 7 (*K7*) staining (*pink*) and proliferative cells were quantified by Ki67 staining (*brown*) (**a**). Double K7/Ki67-positive cells were manually counted (**b**) with the distinction of cells from the ductular reaction (*DR*), intermediate progenitor cells (*iPC*), or intermediate hepatocyte-like cells (*IH*) and K7-negative Ki67-positive cells with a hepatocyte morphology classified as proliferative hepatocytes (*Hep*) (**d**). Morphometric quantification of the area occupied by K7-positive cells is also presented (**c**). All data are shown as mean ± SD. *p < 0.05 between groups

**Fig. 2:**
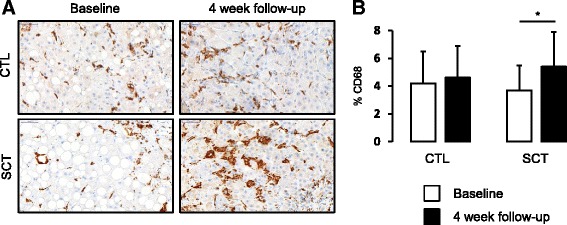
Macrophage compartment analysis at baseline and 4 weeks in controls (*CTL*) and stem cell treated (*SCT*) patients. Macrophages were stained with antibody against CD68 (*brown*) (**a**) allowing the morphometric quantification of the area occupied by those cells (**b**). Data are shown as mean ± SD. *p < 0.05 between groups

At baseline, the control group and SCT group were comparable in terms of histological lesions (Additional file 1). Baseline and 4-week liver biopsies were evaluated with a paired comparison. As reported in our initial paper, patients who received SCT had a similar improvement of liver function over time as the controls, and did not exhibit any increased proliferative activity in K7-positive liver progenitor cells [8]. This proliferating activity, counted on all liver slides with double K7 and Ki67 double immunohistochemistry, even decreased significantly between the baseline biopsy at week 0 and the second biopsy at week 4 (Fig. 1a and b). Hepatocyte (Hep) proliferation also decreased between week 0 and week 4, although not significantly, and was similar between SCT patients and controls (Fig. 1a and b). No significant change in total K7-positive cell area could be seen between week 0 and week 4 and following SCT (Fig. 1c). Double K7-Ki67-positive cells were evaluated in all K7 cell subtypes in the control and SCT patients at week 0 and week 4. The decrease in K7-Ki67-positive cells was significant for proliferative K7^+^ intermediate progenitor cells (iPC), but not for other cell subtypes including cells from the ductular reaction (DR) and intermediate hepatocytes (IH) (Fig. 1d). Furthermore, there was no difference between SCT patients and controls (Fig. 1d). As reported previously [8] and seen on histological sections, steatosis was present at baseline and dramatically decreased after 4 weeks of management (Figs. 1a and 2a, and Additional file 2). This observation is in line with a sustained abstinence from alcohol in the majority of our patients.

Increased macrophage infiltration was identified in our baseline study analysis as a positive prognostic factor. Indeed, improvers show a significantly more important expansion of CD68-positive cells compared to nonimprovers at inclusion [10]. We therefore completed our histological study by exploring whether the liver macrophagic compartment was affected by the stem cell therapy procedure. Both groups (controls and SCT) were comparable at baseline in terms of CD68 cell quantification (Additional file 1). In patients treated with SCT, but not in the control group, we observed a significant increase in the CD68-positive cell compartment at week 4 compared to baseline (Fig. 2a and b). Macrophage content was also higher at 4 weeks in the SCT group compared to the control group, although it did not reach statistical significance (Fig. 2a and b).

Finally, we explored SPINK1 mRNA expression on liver sections, this also being identified to be at higher levels in improvers in the baseline study [10, 16]. SPINK1 mRNA by in situ hybridization was present at week 4 in SCT patients in the liver parenchyma adjacent to macrophage recruitment and liver cell proliferation areas (Additional file 2).

### Effect of SCT on liver gene expression

Further analyses of gene expression patterns were performed in order to determine whether changes in the macrophage compartment were associated or not with any modification in inflammatory and/or regenerative pathways.

SCT was associated with significant changes (defined as fold-change over 2 and *p* < 0.05) in only four transcripts when compared with standard therapy controls at week 4. SPINK1 and the C-X-C motif chemokine ligand (CXCL)6 were upregulated in SCT patients compared to controls, while CXCL14 and glutamate transporter GLT-1 (SLC1A2) were significantly downregulated (Table 2).

**Table 2:** Different expression of genes at the 4-week follow-up in stem cell treated patients compared to control patients

Accession number	Gene name	Affymetrix fold-expression	*p* value	Nanostring fold-expression	*p* value
Macrophages, inflammation, and chemotaxis					
NM_001040059, NM_001251	CD68 CD68 molecule	1.11	0.5874	**5.19**	**0.0011**
NM_002993	CXCL6 (chemokine (C-X-C motif) ligand 6)	**2.03**	**0.0327**	3.42	0.2015
NM_002985	CCL5 (chemokine (C-C motif) ligand 5)	**1.53**	**0.0411**	–	–
NM_033439	IL33 (interleukin 33)	**1.53**	**0.0336**	–	–
NM_000331, NM_001127380, NM_001178006, NM_030754, NM_199161	SAA-1 (serum amyloid A1)	1.73	0.0692	**2.68**	**0.0455**
NM_001127380, NM_030754	SAA-2 (serum amyloid A2)	1.12	0.7873	**3.11**	**0.0202**
NM_004887	CXCL14 (chemokine (C-X-C motif) ligand 14)	**–2.13**	**0.0458**	–6.32	0.1257
Liver regeneration and stem cells					
NM_000601, NM_001010931, NM_001010932, NM_001010933, NM_001010934	HGF (hepatocyte growth factor)	1.11	0.4825	**7.50**	**0.0019**
NM_016639	TNFRSF12A (tumor necrosis factor receptor superfamily, member 12A)	1.15	0.4973	1.08	0.9551
NM_003122	SPINK1 (serine peptidase inhibitor, Kazal type 1)	**4.29**	**0.0446**	19.95	0.0979
NM_005556	CK7(keratin 7)	–1.11	0.7284	–3.38	0.3841
NM_002276	CK19(keratin 19)	1.04	0.8957	–2.15	0.5533
NM_002838, NM_080921, NM_080923	PTPRC (CD45) (protein tyrosine phosphatase, receptor type, C)	**1.52**	**0.0473**	**8.98**	**0.0260**
Fibrosis					
NM_000088	COL1A1 (collagen, type I, alpha 1)	1.38	0.1405	**10.54**	**0.0101**
Other					
NM_001142466, NM_133443	GPT2	**–1.54**	**0.0201**	–	–
NM_001190463, NM_001902, NM_153742	–	**–1.83**	**0.0365**	–	–
NM_001195728, NM_004171	SLC1A2 (solute carrier family 1 (glial high affinity glutamate transporter), member 2)	**–2.22**	**0.0147**	–	–

Values shown in bold are significant at *p* < 0.05

Other significant (*p* < 0.05) changes, but at a lower level (fold-change ranging from 1 to 2), could be observed for CD45, expressed in granulocytes and hematopoietic stem cells, inflammatory chemokine (C-C motif) ligand 5, and interleukin 33, glutamic pyruvate transaminase 2 (GPT2), and cystathionine gamma-lyase (CTH) involved in l-alanine, l-cysteine, and l-methionine metabolism (Table 2). In this context, the three main pathways highlighted by the functional analysis of these transcriptome data using the MetaCore software were cell chemotaxis, the alpha-amino acid metabolic process, and regulation of the antigen receptor-mediating signaling pathway (Additional file 3). This analysis did not reveal any other important pathway and, in particular, no upstream or downstream regulators of SPINK1.

Genes related to the cell cycle, Wnt, or tumor necrosis factor (TNF)-like weak inducer of apoptosis (TWEAK) signaling or liver function were unchanged, consistent with the absence of clinical and biological effects of SCT in patients with AH (Table 2 or data not shown).

Nanostring analysis was then performed on selected genes in order to confirm these results. Interestingly, CD68 expression was significantly upregulated (Table 2), in line with the histological data (Fig. 2). In accordance with this upregulation of inflammation, serum amyloid A1 (SAA-1) was upregulated (Table 2) as was SPINK1 expression, although the increase did not reach statistical significance.

Nanostring analysis also revealed a significant upregulation of hepatocyte growth factor (HGF) and also of collagen type 1 alpha1 (COL1A1) in SCT patients compared to controls at the 4-week liver biopsy. However, careful examination of the genes evolving between baseline and week 4 showed a significant downregulation of COL1A1 in both groups, although this was more pronounced in controls (Table 3). In accordance with the significant decrease in proliferative K7^+^ cells, tumor necrosis factor receptor superfamily member 12A (TNFRSF12A), the receptor for TWEAK (involved in LPC proliferation), was significantly downregulated in controls and SCT patients (Table 3). Other genes related to liver function or metabolism were also significantly modified between baseline and week 4 both in controls and SCT patients, such as detoxification enzyme glutathione S-transferase alpha 1 (GSTA1), cytochrome P450 family 7/1 subfamily A polypetides 1/2 (CYP7A1 and CYP1A2), lipoprotein A (LPA), aldo-ketoreductase family 1, member B10 (AKRB10), fatty acid binding protein 5 (FABP5), and superoxide dismutase 2 (SOD2) (Table 3).

**Table 3:** Different expression of genes at the 4-week follow-up in control patients and in stem cell treated patients compared to baseline

Accession number	Gene name	Control patients				SCT patients			
		Affymetrix fold-expression	*p* value	Nanostring fold-expression	*p* value	Affymetrix fold-expression	*p* value	Nanostring fold-expression	*p* value
Macrophages, inflammation and chemotaxis									
NM_001040059, NM_001251	CD68 CD68 molecule	–1.16	0.0740	–1.65	0.3234	–1.13	0.1404	1.00	0.9935
NM_001130046, NM_004591	CCL20 (chemokine (C-C motif) ligand 20)	**–1.78**	**0.0234**	–	–	**–2.26**	**0.0032**	–	–
NM_003955	SOCS3 (suppressor of cytokine signaling 3)	–1.30	0.1987	–6.5	0.0574	**–1.96**	**0.0033**	**–28.96**	**0.0017**
NM_002228	JUN (jun proto-oncogene)	**–1.65**	**0.0212**	–	–	**–2.07**	**0.0021**	–	–
NM_004887	CXCL14 (chemokine (C-X-C motif) ligand 14)	–1.07	0.7539	–1.39	0.6367	**–1.70**	**0.0278**	**–8.48**	**0.0057**
NM_001100812, NM_022059	CXCL16 (chemokine (C-X-C motif) ligand 16)	**–1.18**	**0.0351**	–**6.37**	**0.0355**	**–1.19**	**0.0342**	**–16.20**	**0.0029**
Liver regeneration, cell cycle and stem cells									
NM_000601, NM_001010931, NM_001010932, NM_001010933, NM_001010934	HGF (Hepatocyte growth factor)	**–1.20**	**0.0529**	–**3.40**	**0.0144**	–1.17	0.1073	–1.32	0.5506
NM_001528.2	HGFAC (Hepatocyte growth factor activator)	1.10	0.4267	–	–	**1.34**	**0.0267**	–	–
NM_001170406, NM_001170407, NM_001786, NM_033379	CDK1 (cyclin-dependent kinase 1)	–1.20	0.3340	–1.19	0.7497	–1.25	0.2553	–1.31	0.6225
NM_031966	CCNB1 (Cyclin B1)	–1.21	0.2251	–2.17	0.2864	–1.38	0.0558	–2.22	0.2748
NM_001255	CDC20 (cell division cycle 20 homolog)	–1.03	0.8235	–1.45	0.5416	–1.18	0.2489	–1.32	0.6487
NM_005556	CK7 (keratin 7)	1.08	0.6855	–2.48	0.3612	–1.20	0.3464	**–12.28**	**0.0178**
NM_002276	CK19 (keratin 19)	–1.16	0.4008	–2.33	0.3545	–1.38	0.0900	**–13.09**	**0.0092**
NM_002838, NM_080921, NM_080923	PTPRC (CD45) (protein tyrosine phosphatase, receptor type, C)	**–1.53**	**0.0098**	–3.61	0.06	–1.04	0.8147	–1.32	0.675
NM_003122	SPINK1 (serine peptidase inhibitor, Kazal type 1)	–2.42	0.0591	–6.19	0.1449	–1.07	0.8789	–1.28	0.8391
NM_016639	TNFRSF12A (tumor necrosis factor receptor superfamily, member 12A)	**–2.02**	**0.0003**	**–11.26**	**0.0207**	**–1.75**	**0.0016**	**–31.33**	**0.0019**
Fibrosis									
NM_000088	COL1A1 (collagen, type I, alpha 1)	**–1.96**	**0.0001**	**–5.41**	**0.0181**	**–1.47**	**0.0137**	–1.34	0.6628
NM_000089	COL1A2 (collagen, type I, alpha 2)	**–2.26**	**0.0001**	**–2.17**	**0.0216**	**–1.61**	**0.0136**	–1.53	0.1871
Metabolism/function									
NM_145740	GSTA1 (glutathione S-transferase alpha 1)	**2.10**	**0.0258**	2.51	0.1955	**2.12**	**0.0296**	**7.85**	**0.007**
NM_000846	GSTA2 (glutathione S-transferase alpha 1)	1.87	0.0573	–	–	**2.72**	**0.0183**	–	–
NM_000780.3	CYP7A1 (cytochrome P450, family 7, subfamily A, polypeptide 1)	**2.72**	**0.0183**	–	–	**2.59**	**0.0193**	–	–
NM_000761	CYP1A2 (cytochrome P450, family 1, subfamily A, polypeptide 2)	**2.15**	**0.0073**	–	–	**2.24**	**0.0067**	–	–
NM_001195728, NM_004171	SLC1A2 (solute carrier family 1 (glial high affinity glutamate transporter), member 2)	**1.38**	**0.064**	–	–	–1.09	0.4444	–	–
NM_005577	LPA (lipoprotein, Lp(a))	**2.18**	**0.0070**	**27.09**	**0.0021**	**2.39**	**0.0041**	**20.77**	**0.0041**
NM_000482	APOA4 (apolipoprotein A-IV)	**–4.56**	**0.0001**	**–17.89**	**0.0053**	**–5.97**	**0.0000**	**–126.73**	0.00001
NM_020299	AKR1B10 (aldo-keto reductase family 1, member B10 (aldose reductase))	**–1.85**	**0.0115**	**–1.61**	**0.0302**	**–2.23**	**0.0024**	**–2.33**	**0.0005**
NM_001444, NR_002935	FABP5/FABP5P3 (fatty acid binding protein 5 (psoriasis-associated)/fatty acid binding protein 5 pseudogene 3)	**–1.65**	**0.0212**	–	–	**–2.07**	**0.0021**	–	–
NM_005764	PDZK1 (PDZK1 interacting protein 1)	–1.49	0.0972	–5.82	0.0655	**–1.73**	**0.0330**	**–12.02**	**0.0124**
NM_000636, NM_001024465, NM_001024466	SOD2 (superoxide dismutase 2, mitochondrial)	**–1.57**	**0.0193**	–1.21	0.2164	**–1.61**	**0.0187**	**–1.44**	**0.0267**

Values shown in bold are significant at *p* < 0.05

## Discussion

Building on our previous report assessing the role of autologous bone marrow stem cell transplantation in AH, we aim to go deeper with this additional analysis in our description of the effect of SCT in AH. Although the invasive strategy that aimed to stimulate regeneration and repair in patients with severe liver failure did not demonstrate clinical benefit over the standard of care treatment, we decided to explore whether specific mechanisms were specifically activated following SCT.

A moderate, although significant, CD68^+^ cell expansion was evident on repeat liver biopsy in patients who received GCSF and SCT as compared to controls. As there is no group with GCSF alone, we are not able to conclude if the increased macrophage content is related to GCSF, to SCT, or to both conditions. This macrophage activation can be interpreted as a negative effect, mainly for two reasons. First, macrophages are part of the innate immune system. Their activation in this situation could be the consequence of the administration of foreign cells originating outside the liver. Indeed, it was speculated by some authors that this macrophage expansion, also evident in rats treated with hepatocyte transplantation, could have a negative impact on the engraftment of the cells [17]. Second, infiltration of inflammatory cells and, in particular, macrophages is the hallmark of early and chronic phases of ALD [18] and nonalcoholic fatty liver disease (NAFLD) [19] where they play a pathogenic role through inflammatory cytokine production and oxidative stress induction. However, CD68^+^ cell expansion can also be interpreted as a positive consequence. Indeed, macrophages (through their secretory functions) are also able to stimulate regeneration [9, 11] and to favor LPC transformation into hepatocytes rather than to cholangiocytes [20, 21]. In summary, heterogeneity exists in macrophage subsets and functions explaining their ambivalent nature. In asymptomatic early phases of ALD or NAFLD, macrophages play a negative role, taking part in the disease process, whereas in late severe stages with massive hepatocellular death (such as this situation of AH), they are able to promote liver regeneration [22]. The identification of one macrophage-derived target mediator (or one specific macrophage population) stimulating liver repair with no or a poor effect on liver injury in AH will be of great interest.

The data on hepatic gene expression changes induced by a single session of SCT were somewhat disappointing, although anticipated in the view of the negative clinical and biological results. Changes in inflammation and chemotaxis-related genes, in SPINK1, and in CD45 hematopoietic cell/granulocyte markers were evident in SCT patients. However, no change in cell-cycle proteins could be seen, a parameter that has been reported as a positive prognostic indicator in previous studies [10, 23]. Nanostring analyses on selected genes revealed a significant upregulation of CD68 (in line with the histological data) and HGF following SCT, and confirmed the upregulation of CD45.

It is possible that the degree of cell engraftment at the site of injury is low, as we were not able to track the bone marrow-derived cells into the liver. The severe inflammatory condition present in AH could also alter this phenomenon. A reduced potential of regeneration for bone marrow stem cells from patients with cirrhosis was also recently described and correlated with the severity of the liver disease assessed by prognostic factors [24]. Another hypothesis is that the bone marrow stimulus in our study (5-day GCSF stimulation followed by one single injection of stem cells in the hepatic artery) was not sufficient to enhance regeneration. In this setting, results of the Realistic study will be of great interest [25]. Indeed, this randomized controlled trial will evaluate the impact of GCSF injections with or without repeated (although peripheral) injections of bone marrow stem cells, compared to controls, in compensated cirrhosis.

## Conclusions

Our results are consistent with a significant but low impact of SCT on hepatic macrophage expansion and on some macrophage/regenerative/stem cell markers. This macrophage activation in AH, concurrent with higher SPINK1 and HGF mRNA expression in SCT patients, is thus analyzed as a positive factor. However, with the negative results of the clinical trial, it has to be interpreted as a weak impact of stem cell transplantation, which is not able to modify the clinical course of the severe disease. Dysfunction of bone marrow stem cells in severe cirrhosis and the difficulties of engraftment of transplanted cells in a severe target disease in this context of decompensated alcoholic cirrhosis represent two possibilities that might explain the low impact of this SCT strategy. The strengths of this study are the high number of patients included in the histological and transcriptome analysis, as well as the baseline and 4-week liver material, allowing a concordance between the results and the clinical impact of SCT.

## Additional files

Additional file 1: Baseline histological patient characteristics according to treatment allocation. (PDF 105 kb)

Additional file 2: Liver cell proliferation, macrophage activation, and SPINK1 mRNA expression. This figure shows the expression by immunohistochemistry of K7 (pink), Ki67 (brown) (A) and CD68 (brown) (B), and the mRNA expression of SPINK1 (blue) revealed by in situ hybridization (C). One control patient (CTL) and two different stem cell treated (SCT) patients are illustrated with serial sections at 4 weeks. SPINK1 mRNA positivity could be observed in the liver parenchyma of SCT patients at week 4. (PPTX 2667 kb)

Additional file 3: Top 3 regulatory gene ontology processes in stem cell treated (SCT) patients identified by MetaCore analysis system at 4 weeks. Using a fold-change threshold of 1.5 and a *p* value lower than 0.05, three sets of biological processes were identified (based on *p* value). A red color next to the gene symbol represents significantly upregulated genes while a green color represents significantly downregulated genes in SCT patients compared to controls. (PPTX 52 kb)

## Abbreviations

AH: Alcoholic hepatitis
ALD: Alcoholic liver disease
COLA1A1: Collagen type 1 alpha1
CXCL: C-X-C motif chemokine ligand
DR: Ductular reaction
GCSF: Granulocyte-colony stimulating factors
Hep: Hepatocyte
HGF: Hepatocyte growth factor
IH: Intermediate hepatocytes
iPC: Intermediate liver progenitor cells
K7: Keratin 7
LPC: Liver progenitor cell
MELD: Model for end-stage liver disease
NAFLD: Nonalcoholic fatty liver disease
SCT: Stem cell therapy
SPINK1: Serine peptidase inhibitor Kazal type 1
TNFRSF12A: Tumor necrosis factor receptor superfamily member 12A
TWEAK: Tumor necrosis factor-like weak inducer of apoptosis
