# Supplementary material for: Autologous bone marrow-derived cell transplantation in decompensated alcoholic liver disease: what is the impact on liver histology and gene expression patterns?
Source: Stem Cell Res Ther. 2017 Apr 18;8:88. doi: 10.1186/s13287-017-0541-2 (PMC5395856; doi:10.1186/s13287-017-0541-2)
Supplement: Supplementary file 1 — Baseline histological patient characteristics according to treatment allocation. (PDF 105 kb) [file 13287_2017_541_MOESM1_ESM.pdf]

Additional file 1

Baseline histological patient characteristics according to treatment allocation

Variable	Controls n=30	Stem cell transplantation n=28	P value
Steatosis grade (points)	2.3 ± 1.9	2.4 ± 0.8	0.47
Hepatocyte ballooning score (points)	1.4 ± 0.6	1.8 ± 0.5	0.13
Lobular inflammation (points)	1.7 ± 0.8	1.8 ± 0.9	0.58
Portal inflammation (points)	0.7 ± 0.6	0.7 ± 0.5	0.92
Cirrhosis (%)	100	100	0.99
K7 total (%)	8.2 ± 6.8	6.7 ± 6.7	0.41
K7-Ki67 DR (n/field)	0.5 ± 0.6	0.6 ± 0.6	0.68
K7-Ki67 iPC (n/field)	0.7 ± 0.6	0.8 ± 0.7	0.73
K7-Ki67 IH (n/field)	0.2 ± 0.2	0.3 ± 0.3	0.38
Hep Ki67 (n/field)	2.6 ± 2.2	3.8 ± 3.7	0.16
K7-Ki67 total	1.4 ± 1.3	1.6 ± 1.4	0.49
CD68 (%)	4.2 ± 2.3	3.7 ± 1.8	0.38

Abbreviations: keratin 7 (K7), ductular reaction (DR), intermediate progenitor cells (iPC), intermediate hepatocyte like cells (IH), hepatocytes (Hep).
