# Supplementary figures and images for: Autologous bone marrow-derived cell transplantation in decompensated alcoholic liver disease: what is the impact on liver histology and gene expression patterns?
Source: Stem Cell Res Ther. 2017 Apr 18;8:88. doi: 10.1186/s13287-017-0541-2 (PMC5395856; doi:10.1186/s13287-017-0541-2)

## Slide 1
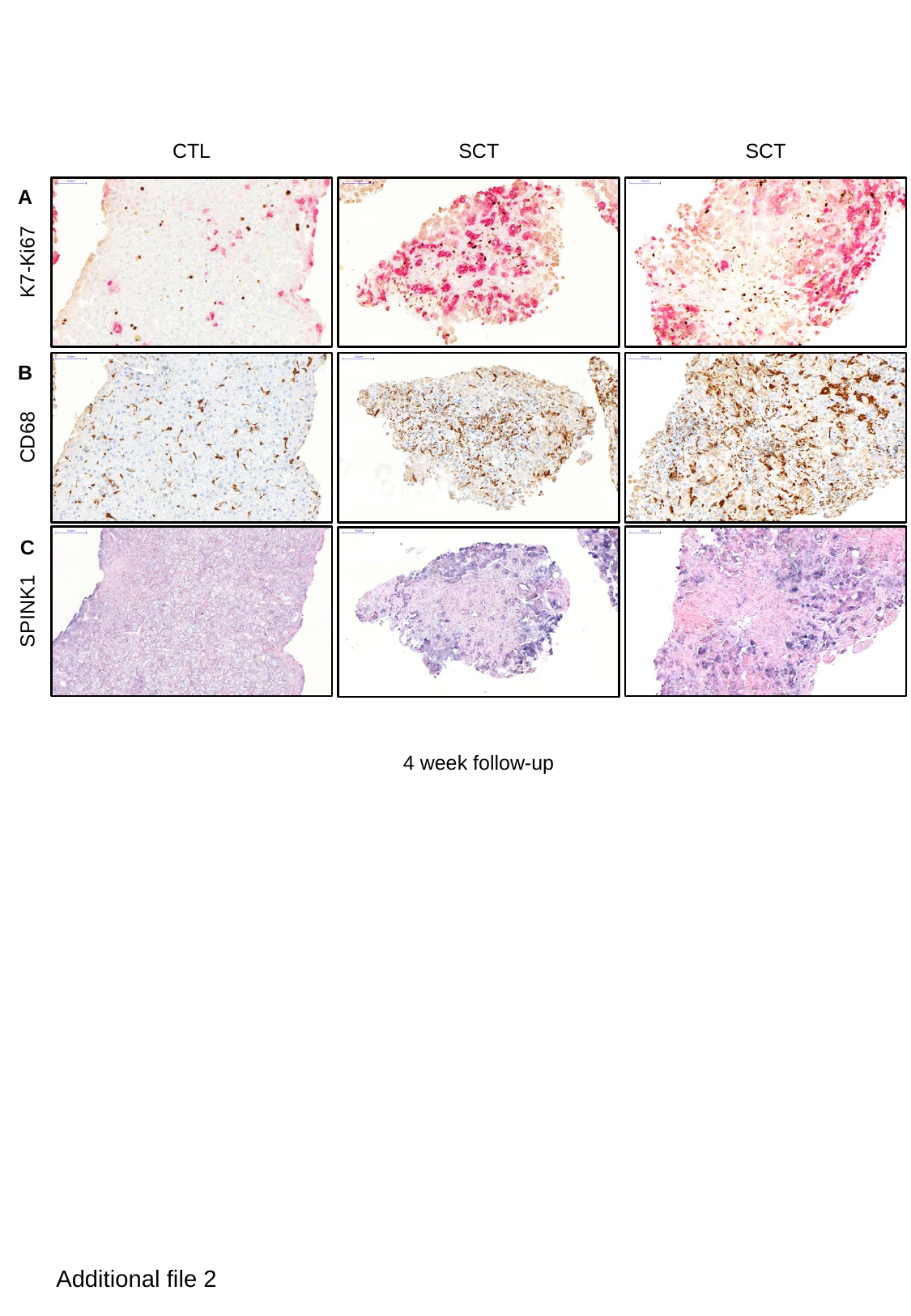

CTL
SCT
SCT
A
K7-Ki67
B
CD68
C
SPINK1
4 week follow-up
Additional file 2

Supplement: Supplementary file 2 — Liver cell proliferation, macrophage activation, and SPINK1 mRNA expression. This figure shows the expression by immunohistochemistry of K7 (pink), Ki67 (brown) (A) and CD68 (brown) (B), and the mRNA expression of SPINK1 (blue) revealed by in situ hybridization (C). One control patient (CTL) and two different stem cell treated (SCT) patients are illustrated with serial sections at 4 weeks. SPINK1 mRNA positivity could be observed in the liver parenchyma of SCT patients at week 4. (PPTX 2667 kb) [file 13287_2017_541_MOESM2_ESM.pptx]
