# Supplementary material for: Autologous bone marrow-derived cell transplantation in decompensated alcoholic liver disease: what is the impact on liver histology and gene expression patterns?
Source: Stem Cell Res Ther. 2017 Apr 18;8:88. doi: 10.1186/s13287-017-0541-2 (PMC5395856; doi:10.1186/s13287-017-0541-2)
Supplement: Supplementary file 3 — Top 3 regulatory gene ontology processes in stem cell treated (SCT) patients identified by MetaCore analysis system at 4 weeks. Using a fold-change threshold of 1.5 and a p value lower than 0.05, three sets of biological processes were identified (based on p value). A red color next to the gene symbol represents significantly upregulated genes while a green color represents significantly downregulated genes in SCT patients compared to controls. (PPTX 52 kb) [file 13287_2017_541_MOESM3_ESM.pptx]

## Slide 1
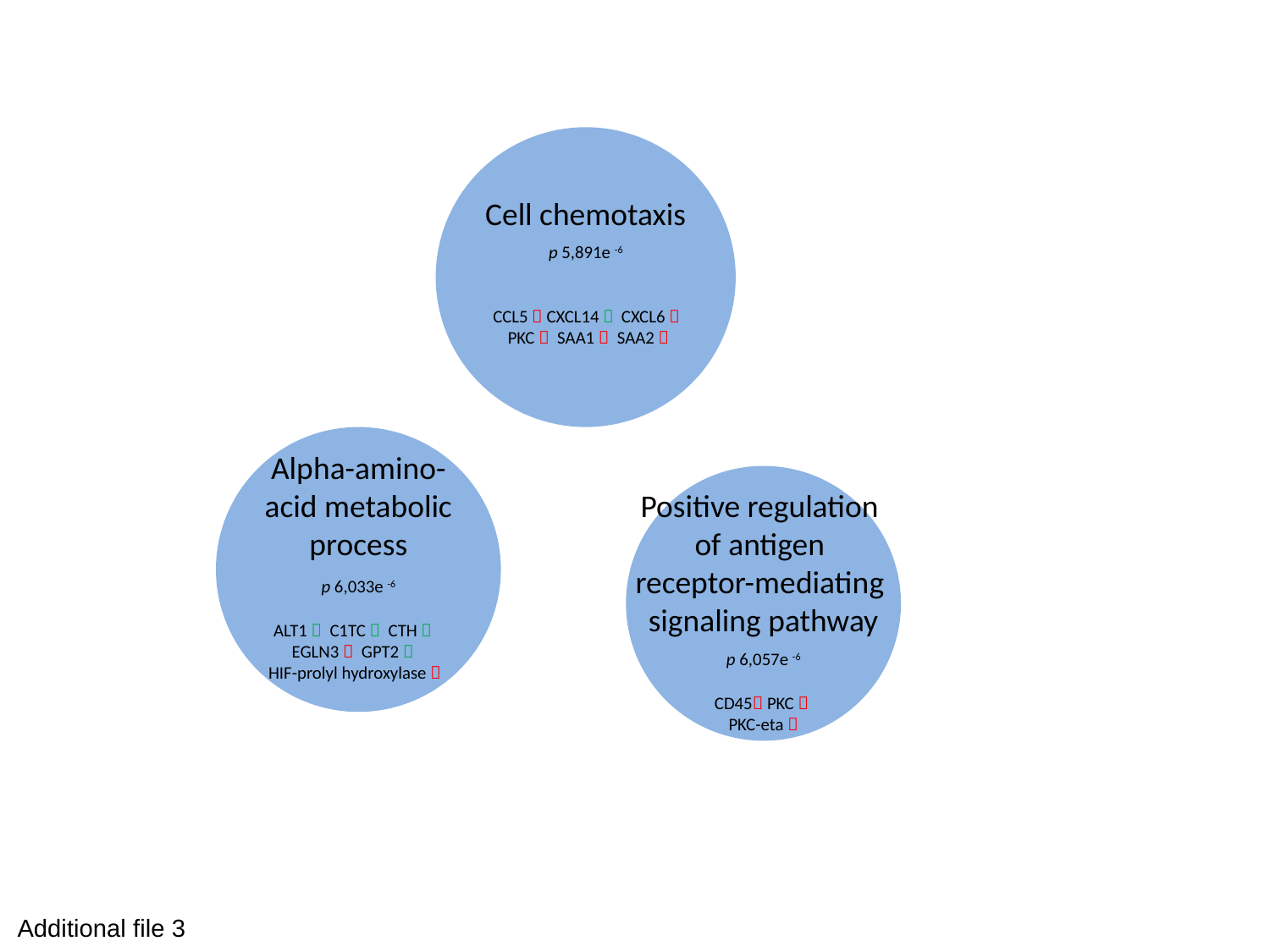

Cell chemotaxis
p 5,891e -6
CCL5 γ CXCL14 γ CXCL6 γ
PKC γ SAA1 γ SAA2 γ
Alpha-amino-acid metabolic process
Positive regulation
of antigen
receptor-mediating
signaling pathway
p 6,033e -6
ALT1 γ C1TC γ CTH γ
EGLN3 γ GPT2 γ
HIF-prolyl hydroxylase γ
p 6,057e -6
CD45γ PKC γ
PKC-eta γ
Additional file 3
